# Prognostic Impact of Sarcopenia’s Occurrence during Radiotherapy in Oropharyngeal Cancer Patients

**DOI:** 10.3390/cancers15030723

**Published:** 2023-01-24

**Authors:** Luca Bergamaschi, Giulia Marvaso, Mattia Zaffaroni, Maria Giulia Vincini, Oriana D’Ecclesiis, Stefania Volpe, Annamaria Ferrari, Stefano Filippo Zorzi, Maria Cossu Rocca, Annarita Sabbatini, Giulia Cannillo, Emanuela Zagallo, Anna Starzyńska, Mohssen Ansarin, Federica Cattani, Sara Gandini, Roberto Orecchia, Daniela Alterio, Barbara Alicja Jereczek-Fossa

**Affiliations:** 1 Division of Radiation Oncology, European Institute of Oncology IRCCS, 20141 Milan, Italy; 2 Department of Oncology and Hemato-Oncology, University of Milan, 20122 Milan, Italy; 3 Department of Experimental Oncology, European Institute of Oncology, IRCCS, 20141 Milan, Italy; 4 Department of Otolaryngology and Head and Neck Surgery, European Institute of Oncology IRCCS, 20141 Milan, Italy; 5 Department of Medical Oncology, Urogenital and Head and Neck Tumors Medical Treatment, European Institute of Oncology IRCCS, 20141 Milan, Italy; 6 Dietetic and Clinical Nutrition Unit, European Institute of Oncology IRCSS, 20141 Milan, Italy; 7 Department of Oral Surgery, Medical University of Gdańsk, 80210 Gdańsk, Poland; 8 Unit of Medical Physics, European Institute of Oncology, IRCCS, 20141 Milan, Italy; 9 Scientific Directorate, European Institute of Oncology, IRCCS, 20141 Milan, Italy

**Keywords:** sarcopenia, oropharyngeal carcinoma, HPV, oncological outcomes, nutritional support

## Abstract

**Simple Summary:**

Several retrospective studies have shown worse oncological outcome and toxicity in sarcopenic patients with head and neck cancer undergoing radiotherapy. The current study focuses on patients with oropharyngeal carcinoma, especially HPV-associated, to investigate whether sarcopenia, not only present at baseline before starting treatment, but also arising during radiotherapy, could impact oncological outcomes and toxicity in these good-prognosis patients. Indeed, the intensification of a tailored approach with prompt nutritional intervention, trying to intercept patients before the onset of sarcopenia, could improve oncological outcomes.

**Abstract:**

The current study aims to profile sarcopenic condition (both at baseline and developed during treatment) in oropharyngeal carcinoma (OPC) patients treated with curative radiotherapy (RT) +/− chemotherapy and to evaluate its impact on oncological outcomes and toxicity. A total of 116 patients were included in this retrospective single-center study. Sarcopenia assessment at baseline and at 50 Gy re-evaluation CT was obtained from two different methodologies: (i) the L3-skeletal muscle index (SMI) derived from the contouring of the cross-sectional area (CSA) of the masticatory muscles (CSA-MM); and (ii) the paravertebral and sternocleidomastoid muscles at the level of the third cervical vertebra (CSA-C3). Based on L3-SMI from CSA-MM, developing sarcopenic condition during RT (on-RT sarcopenia) was associated with worse progression-free survival (PFS) (*p* = 0.03) on multivariable analysis and a trend of correlation with overall survival (OS) was also evident (*p* = 0.05). According to L3-SMI derived from CSA-C3, on-RT sarcopenia was associated with worse PFS (*p* = 0.0096) and OS (*p* = 0.013) on univariate analysis; these associations were not confirmed on multivariable analysis. A significant association was reported between becoming on-RT sarcopenia and low baseline haemoglobin (*p* = 0.03) and the activation of nutritional counselling (*p* = 0.02). No significant associations were found between sarcopenia and worse RT toxicity. Our data suggest that the implementation of prompt nutritional support to prevent the onset of sarcopenia during RT could improve oncological outcomes in OPC setting.

## 1. Introduction

The incidence of oropharyngeal carcinoma (OPC) is increasing in recent years [1], especially due to the role played by the human papillomavirus (HPV) in triggering its development [2,3]. Patients with HPV-associated tumors are more often male, young, fit, non-smokers, with good dental condition, with good social and economic status and, indeed, with a better prognosis [4]. However, current guidelines do not provide deintensification protocol in HPV+ patients [5,6]. A promising field of research concern radiotherapy (RT) deintensification studies, aimed at identifying new personalized RT schedules in the case of HPV-associated OPC [7,8,9,10,11]. Sarcopenia is a condition of reduced total muscle mass of the body, associated with a decrease in function [12] and often reported in oncological patients [13,14]. Sarcopenic condition is usually assessed based on a single-slice computed tomography (CT) measurement of the cross-sectional area (CSA) of muscles at the level of the third lumbar vertebra (CSA-L3) [15,16]. According to several published studies, sarcopenia seems to be associated with a worse prognosis, worse tolerance to the proposed treatments [13,14,17,18,19], and enhanced inflammatory status [16,20,21,22,23,24,25]. In patients with Head and Neck (H&N) tumors, sarcopenia is frequently reported, due to both the local extension of the neoplasia, that often compromises adequate nutrition (sarcopenic condition at baseline, before starting RT), and the tolerance to proposed treatments, such as RT, that can worsen malnutrition in patients with normal baseline muscle mass (sarcopenic condition developed during RT) [26,27,28,29,30,31]. Indeed, in H&N cancer patients, RT could lead to skeletal muscle loss and to the onset of both acute and late toxicities, which can result in inadequate oral feeding and trigger sarcopenia [18,31,32,33,34]. Moreover, RT toxicities can worsen when combined treatment with systemic agents is prescribed. In patients with H&N cancer, sarcopenia is often assessed by contouring the CSA of the paravertebral and sternocleidomastoid muscles at the level of the third cervical vertebra (CSA-C3) [26,30,35,36,37,38,39]. More recently, the contouring of the CSA of the masticatory muscles (CSA-MM) has also been proposed as a valid method to estimate sarcopenic condition [40]. Since, as of today no standardization exist for sarcopenia assessment, both methods will be tested in the present study, in order to evaluate the impact of the two approaches for identification of the sarcopenic condition. The main aim of the study is to investigate the impact of sarcopenia (at baseline or developed during RT treatment) on oncological outcomes and RT toxicities in a relatively fit population of OPC patients treated with RT+/− chemotherapy (ChT). This is essential in developing personalized and multidisciplinary preventive approaches, such as prompt baseline nutritional support for each patient to avoid the onset of sarcopenia during RT, thus preventing its eventual negative impact.

## 2. Materials and Methods

Patients treated with RT at the Department of Radiation Oncology of the European Institute of Oncology IRCCS (IEO), Milan, Italy from 2012 to 2019 were retrospectively considered for study inclusion.

### 2.1. Eligibility Criteria

Inclusion criteria were as follows:

(i). Age more than 18 years;
(ii). Confirmed primary tumor of the oropharynx with histological diagnosis of squamous cell carcinomas;
(iii). Patients treated with curative intent;
(iv). Patients treated with RT with volumetric modulated arc therapy (VMAT) technique, either combined or not with systemic treatment;
(v). The availability of simulation CT (sim-CT) images;
(vi). Written informed consent signed for research purpose.

Exclusion criteria:

(i). Patients treated with postoperative adjuvant or palliative RT;
(ii). Patients with distant metastases or second synchronous tumors.

### 2.2. Clinical Parameters

**Figure 1:**
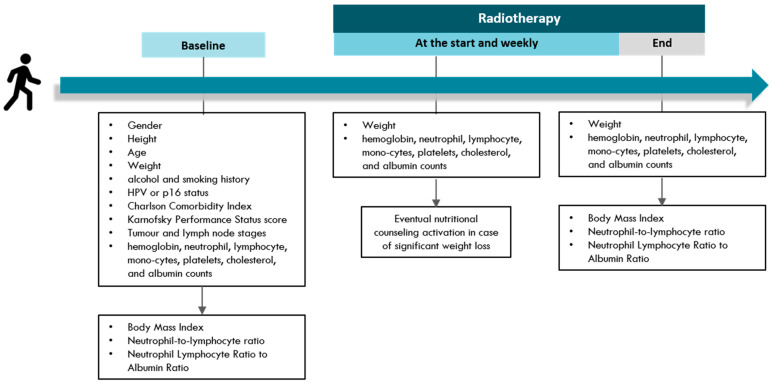
Summary of collected clinical parameters at time points of interest.

Clinical parameters including gender, height, age, alcohol and smoking history, HPV or p16 status, Charlson Comorbidity Index (CCI), and Karnofsky Performance Status (KPS) score were retrospectively collected. Weight and blood count parameters were collected both at baseline and at the end of RT. Body Mass Index (BMI) and Neutrophil-to-lymphocyte ratio (NLR) were calculated at baseline and end of RT, as well. A summary of clinical parameters and time points of interest is provided in Figure 1.

Tumor and lymph node stages were defined according to the 7th edition of the American Joint Committee on Cancer/Union for International Cancer Control (AJCC/IUCC).

Physician-rated RT-related acute and late toxicities (according to the Common Terminology Criteria for Adverse Events (CTCAE) version 4.0 [41]), date of last follow-up, and the eventual date of recurrent disease or death, were obtained from patients’ records.

### 2.3. Treatment Characteristics

All patients had a sim-CT scan with and without administration of intravenous contrast medium, in a supine position and with a thermoplastic mask to immobilize head and shoulders. All CT-scans were acquired with GE Healthcare Optima CT580 W scanner and with the same acquisition protocol (120-kV tube voltage, 150-mA tube current, and 2.5-mm slice thickness). As a standard procedure, all patients underwent a routine dental check-up before the treatment. Curative RT was administered with VMAT and simultaneous integrated boost (SIB) technique (70 Gy, 2 Gy/fx; 63 Gy, 1.8 Gy/fr; 58.1 Gy, 1.66 Gy/fr) in 35 fractions. During RT, as standard procedure, patients underwent a re-evaluation scan (rev-CT) at 40, 50, and 60 Gy without contrast administration. Concomitant systemic treatment was also prescribed, except for selected cases of early-stage disease or for patients with contraindications. Concomitant treatments involved the administration of cisplatin 100 mg/m^2^ every 3 weeks for 3 times, weekly cisplatin 40 mg/m^2^, weekly carboplatin, weekly cetuximab, and neoadjuvant ChT with docetaxel, cisplatin, and 5-fluorouracil (TPF) followed by concurrent cisplatin [42,43,44,45]. All patients were treated on an outpatient basis with Day Hospital access for ChT only, and regular weekly physician and nurse check was performed in order to monitor toxicity, nutritional status, early tumor outcome, and overall treatment compliance.

### 2.4. Sarcopenia Assessment

**Figure 2:**
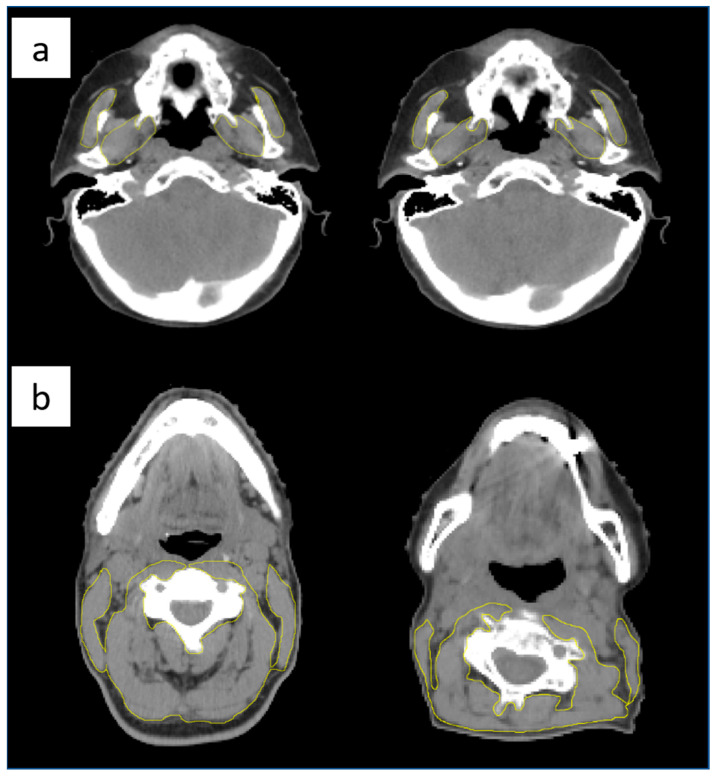
Axial CT-scan image delineation of the masticatory muscles (**a**) and paravertebral and sternocleidomastoid muscles at the level of the third cervical vertebra (**b**) in a non-sarcopenic patient (**left**) and in a sarcopenic patient (**right**).

Sarcopenia was assessed using the L3-Skeletal Muscle Index (SMI), estimated according to previously published formulas [35,40]. For each available sim-CT and rev-CT at 50 Gy, a single radiation oncologist performed the contouring of the cross-sectional areas (CSA) of:

- Masticatory muscles (MM): identified as the masseter and the pterygoid muscles, taken on the first CT slice showing the bilateral mandibular notches when scrolling from caudal to cranial direction (Figure 2a) [40]. The CSA of MM (CSA-MM) was used to estimate the L3-SMI, based on the model proposed by Chang et al. [40] and sarcopenia was then defined using the sex-specific cut off points for L3- SMI of <38.5 cm^2^/m^2^ for women and <52.4 cm^2^/m^2^ for men, as described by Prado et al. [46].
- Paravertebral and sternocleidomastoid muscles: taken at the level of the third cervical vertebra (C3), at the first CT slice identifying the entire vertebral arch of C3 when scrolling from caudal to cranial direction (Figure 2b). The CSA of these muscles at the level of C3 (CSA-C3) was then used to estimate the L3-SMI based on the formula validated by Swartz et al. [35]. Sarcopenia was then defined using the proposed sex-specific cut-off points for L3- SMI of <30.6 cm^2^/m^2^ for women and <42.4 cm^2^/m^2^ for man by Van Rijn-Dekker et al. [30].

Contouring was performed using the freeware package ImageJ (version 1.52a; Wayne Rasband, National Institutes of Health, Bethesda, MD, USA), setting a radiodensity range from −29 up to +150 Hounsfield units to avoid over- or under-estimation of the muscle area.

### 2.5. Outcomes of Interest

Primary outcomes of the study were the association between sarcopenia and progression-free survival (PFS) and overall survival (OS). Secondary outcomes were the association between sarcopenia and physician-rated RT-related toxicities during RT, at the end of RT and after one year from the end of RT, blood parameters at baseline and at the end of RT and the activation of nutritional consultation during RT. PFS was defined as the time from the end of RT until the first occurrence of any of the following events: locoregional progression, systemic progression, or death. OS was calculated from the end of RT to the date of death or last follow-up. All patients alive or free of progression at the last follow-up date were considered right censored.

### 2.6. Statistical Analysis

For continuous variables median and interquartile range (IQR) were reported, absolute and relative frequencies were assessed as summary measures of categorical variables. Based on the nature of variables, Fishers-Exact tests and Wilcoxon Rank tests were performed to investigate associations of sarcopenia status assessed with two methodologies and at different time points, with clinical characteristics, blood parameters and RT toxicity. The Mantel-Haenszel Chi-square test for dose-response was used to assess the association with toxicity grade.

PFS and OS were estimated with the Kaplan-Meier (KM) method and survival distributions were compared using Log-Rank test. Multivariable Cox proportional hazard model was used to determine the the independent prognostic role of sarcopenia with cancer progression or death, adjusting for other prognostic factors and confounders. The significance level was set at a global 2-tailed *p*-value of <0.05 for all analyses. The statistical analyses were performed with Rstudio software, version 4.1.1.

### 2.7. Ethical Considerations

This study complied with the principles of the Declaration of Helsinki and was approved by the IEO IRCCS Ethical Committee (Notification No. UID 3544). All CT image files and clinical records were anonymized and only patients with written informed consent signed for research purposes were included.

## 3. Results

### 3.1. Patients’ and Treatment Characteristics

A total of 116 patients met the inclusion criteria and were included in the study. The median age of was 60 years (IQR 53–67) and the median follow-up was 61.2 months (range 36.3–84.9). A summary of the patients’ characteristics is reported in Table 1.

Among them, 105 (90.5%) underwent combined treatment of RT and chemotherapy (CRT) with curative intent, while 11 patients (9.5%) received indication for exclusive RT. The median overall treatment time was 54 days (IQR 51–58). When concomitant CRT was performed, cisplatin 100 mg/m^2^ every 3 weeks for three administrations was prescribed in 72 cases (68.6%). Other systemic therapies included: weekly cetuximab (16 patients), neoadjuvant ChT with TPF followed by concurrent cisplatin (eight patients), weekly cisplatin (seven patients), and weekly carboplatin (two patients). Regarding RT treatment, 103 patients received 70 Gy as prescribed (88.8%), 12 patients received less than 70 Gy due to excessive toxicity or comorbidities (i.e., systemic lupus erythematosus), and one patient received 72 Gy, as a compensation for a long period of RT interruption. One patient discontinued RT at 50 Gy due to excess toxicity, 20 patients had an interruption during treatment (up to a maximum of 30 days), whereas 95 did not interrupt RT. Nutritional counselling was required during RT for 31 patients (26.3%). The insertion of a nasogastric tube and the placement of a percutaneous endoscopic gastrostomy (PEG) occurred in 14 (12.1%) and 12 patients (10.3%), respectively. The 50 Gy rev-CT was available for 108 patients (93%).

### 3.2. Sarcopenic Condition at Baseline

At baseline, according to CSA-MM method, 24 out of 116 patients were found to be sarcopenic (20.7%). No patients were found to be sarcopenic at baseline according to CSA-C3 method.

At univariate analysis (Supplementary Materials, Table S1), baseline sarcopenia resulted associated with male sex (*p* = 0.0008) and with HPV-not-associated carcinoma (*p* = 0.04).

KM curves for PFS and OS showed no significant difference between patients with and without baseline sarcopenia (Supplementary Materials, Figure S1), and this finding was confirmed at multivariable analysis (Supplementary Materials, Table S2).

### 3.3. Sarcopenic Condition Developed during RT

#### 3.3.1. According to L3-SMI Derived from CSA-MM

**Figure 3:**
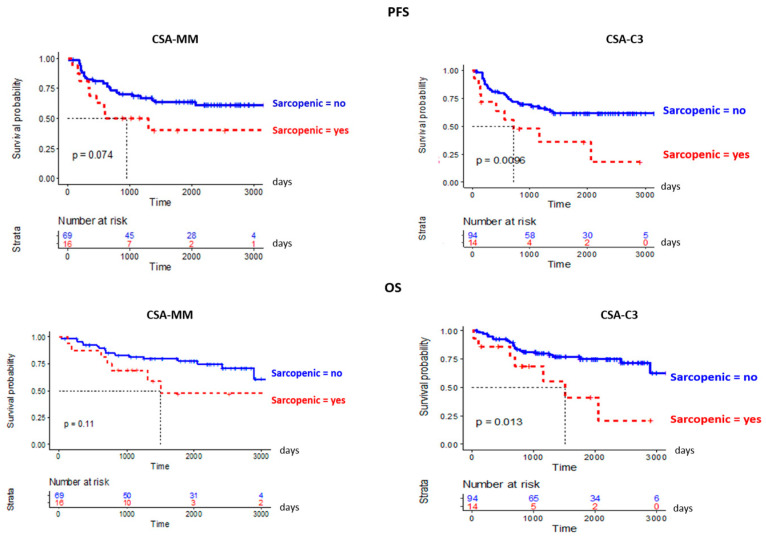
KM curves for OS and PFS of patients become sarcopenic during RT vs. never sarcopenic, based on MM-CSA and C3-CSA.

A 50 Gy rev-CT was available for 108 patients (93%). Of them, according to L3-SMI derived from CSA-MM, 39 out of 108 patients (15%) resulted sarcopenic at 50 Gy evaluation. Among them, 16 patients were not sarcopenic at baseline. Comparing this subgroup of patients who developed sarcopenia during RT (on-RT sarcopenia) with never-sarcopenic patients, on univariate analysis (Supplementary Materials, Table S3), the onset of sarcopenia was significantly associated with male sex (*p* = 0.007) and with HPV-not associated carcinoma (*p* = 0.007). KM curves for PFS and OS are shown in Figure 3. There was no significant difference in PFS and OS between the two groups, however a trend of correlation of on-RT sarcopenia with worse PFS was present (*p* = 0.074), confirmed on multivariable analysis (*p* = 0.03).

On multivariable, a significant association of sarcopenic condition, male sex, high baseline hemoglobin values, and nasogastric tube placement was found both for worse OS and PFS. A worse OS was significantly associated with increased value of CCI, also, while worse PFS with KPS < 100 and increased NLR at baseline. Hazard ratios with 95% CI estimates from multivariable analyses are shown in Table 2. Same results were found when muscle mass loss at 50 Gy was considered as continuous variable (Table S9).

No significant associations were found on univariate analysis between sarcopenia development and blood parameters collected both at baseline and at the end of RT (Supplementary Materials, Table S4), as well as nutritional counselling activation (Supplementary Materials, Table S5) and RT toxicity during, at the end, and one year after the treatment.

#### 3.3.2. According to L3-SMI Derived from CSA-C3

According to L3-SMI derived from CSA-C3, 14 out of 108 patients were found to be sarcopenic at 50 Gy (13%). Comparing this subgroup with never-sarcopenic patients, on-RT sarcopenia resulted associated with older age (*p* < 0.001) and with low baseline haemoglobin values (*p* = 0.03) on univariate analysis (Supplementary Materials, Table S6). KM curves for PFS and OS are shown in Figure 3. A significant difference between the two groups was found both for PFS (*p* = 0.0096) and OS (*p* = 0.013). On multivariable analysis, worse PFS and OS did not result associated with on-RT sarcopenia. A significant association with worse PFS and OS was found for male sex, nasogastric tube placement and high baseline hemoglobin values. OS resulted significantly associated also with alcohol history, KPS < 100, and high NLR at baseline (≥3). Risk estimates from multivariable analysis are shown in Table 2. At univariate analysis, a significant association was found between on-RT and reduced baseline hemoglobin values (*p* = 0.03) (Supplementary Materials, Table S7). No other significant association was found with blood parameters collected both at baseline and at the end of RT.

Finally, a significant association was found between on-RT sarcopenia and the activation of nutritional counselling during RT (*p* = 0.02) (Supplementary Materials, Table S8). Indeed, 57.1% of sarcopenic patients underwent nutritional counselling activation during RT. No significant associations were found between on-RT sarcopenia and RT toxicity during, at the end, and one year after the treatment.

## 4. Discussion

In recent years, sarcopenia has been a hot topic with a growing interest in scientific research in different oncological settings, in order to identify its impact on outcomes and treatment toxicities [14,47]. Different studies have been conducted specifically for H&N cancers, considering the severe malnutrition that is often reported, due to the neoplasia itself and the therapies prescribed [18,32]. In particular, RT alone, or even more with ChT, is associated with the onset of different acute sequelae, such as mucositis and dysphagia, which affect an adequate dietary intake and compromise QoL during RT [32].

In the present work, the impact of sarcopenia was assessed in a specific population of patients with OPC treated with curative RT +/− concomitant ChT.

In particular, two different methodologies with relative cut-off values for sarcopenia identification were tested to evaluate their impact on the sarcopenic condition identification at two different time points (baseline and at the 50 Gy rev-CT). In our cohort, baseline sarcopenia was not related to worse oncological outcome nor to increased RT toxicity. These results suggest that the chosen thresholds may not be optimal for our peculiar population of OPC patients. Conversely, on-RT sarcopenia was associated with worse OS and PFS, considering both models of sarcopenia assessment, while no correlation with toxicities during or after the end of treatment was found.

These results are in line with the growing body of literature reporting the association between sarcopenia and the aforementioned outcome of interest.

In 2020, Findaly et al. published a systematic review of the literature regarding the topic of sarcopenia in patients with H&N cancer, focusing on studies that assessed sarcopenia from CT images and evaluated its association with outcomes [48]. They concluded that sarcopenia is an independent prognostic factor for OS and treatment completion and that further research is essential to improve knowledge about this association and to enable future personalized approach with nutritional support.

A summary of the most recent published studies on sarcopenia in H&N cancer is available in Table 3.

These are all retrospective studies considering patients with H&N carcinomas as a single category [16,27,30,50,51,52,53,54]. Conversely, the current study evaluates sarcopenia only in patients with OPC, mostly HPV-associated. In the case of HPV-associated carcinomas, patients have a good performance status and a better prognosis when RT is administered [4]. For these reasons, deintensification studies were conducted, in order to reduce the impact on QoL of these patients [7,8,9,10,11]. Indeed, a recent systematic review published by Edwards et al. investigated the role of malnutrition in patients with HPV-associated OPC, arguing how research in this field is still limited and specific studies are needed [55]. In fact, a greater weight loss and higher utilization of reactive feeding tubes and lower feeding tube dependency rates were reported for patients with HPV-associated OPC, even if with low certainty grade. Furthermore, it was unclear whether nutritional intake and nutritional status differed between HPV-associated or not subgroups. In addition, an appealing study published by Olson et al. wondered about the best treatment for sarcopenic patients with OPC, concluding that surgery might be the treatment of choice, considering the association between sarcopenia and worse outcomes after RT [56]. In this setting, our effort to assess whether sarcopenia can be easily detected in OPC patients’ subpopulation may play a role in proposing personalized, more effective, and more tolerated treatments.

**Figure 4:**
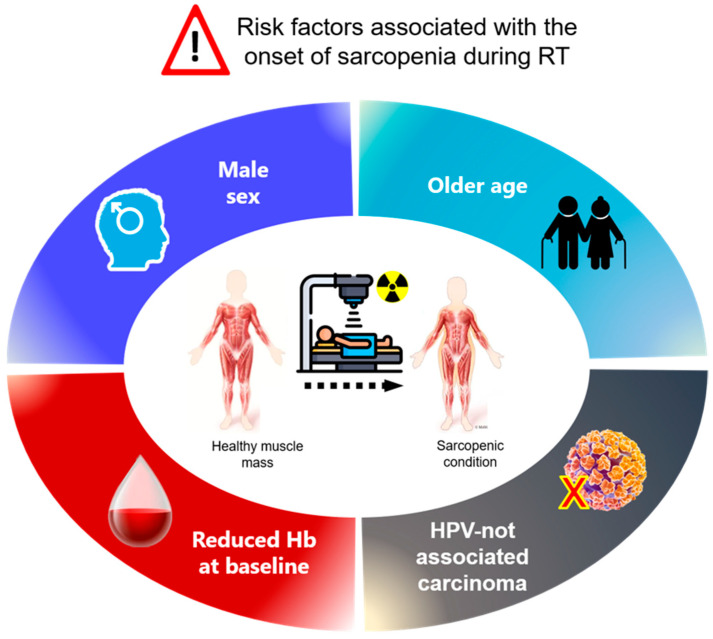
Profile of the patient most at risk for the onset of sarcopenia during radiotherapy.

Since it is not always clear which patients are most likely to become sarcopenic during RT, providing prompt nutritional counselling to all H&N patients or identifying factors that correlate with an increased risk of on-RT sarcopenia (Figure 4) could be useful strategies to intercept patients before the onset of sarcopenia and avoid its detrimental influence. Indeed, patients already sarcopenic at baseline usually are easier identified even before starting RT and therefore could benefit from an early activated nutritional support [57]. Conversely, patients becoming sarcopenic during RT are usually followed up with less intensive nutritional support. Regarding this topic, preliminary data from recent studies seem to show that personalized nutritional support may benefit H&N patients undergoing RT reducing the impact of sarcopenia, through increased calories and protein intake and the use of modulators of the immunologic response [58,59].

The majority of the studies evaluating sarcopenia in H&N cancers depend on the contouring of the paravertebral and sternocleidomastoid muscles at the C3 level, relying on an effective correlation between CSA-C3 and L3-CSA [26,30,35,36,37,39,60]. However, this correlation is no longer so well-established [49,61]; in particular, in locally advanced H&N disease, the presence of lymphadenopathies is common at the level of these muscles and the risk of overestimation of the CSA-C3 contouring is consistent [40].

The second model tested on our sample involves the contouring of the CSA-MM (masseter and pterygoids muscles). Although its use is less frequent, it could be useful to implement it, considering the lower bias in the contouring [40]. We decided to test both methodologies to evaluate their intrinsic differences since, as of today, no standardized and univocal validated methodology for sarcopenia identification is available. In addition, two different cut-off values were tested to evaluate their intrinsic differences in sarcopenia assessment. The cut-off relative to CSA-C3 proposed by Van Rijn-Dekker et al. [30] is derived from the lowest gender-specific quartile in their population, with a mean baseline BMI similar to the one of the present population (25.6 kg/m^2^ vs. 26.01 kg/m^2^). Conversely, CSA-MM cut-off values proposed by Prado et al. [46] through optimal stratification derived from an obese population (mean BMI 34.3 kg/m^2^); this choice was made to take into account the fact that our population was mainly composed of male young patients with HPV-associated OPC, which often represents a particularly fit subset when compared with other H&N settings.

While previous studies focused on the assessment of baseline sarcopenia [16,26,30,40,50,51,52,53,54], another peculiarity of the present work is represented by the assessment of sarcopenia on the rev-CTs at 50 Gy.

In this way, it was possible to investigate the onset or the worsening of sarcopenia during RT, thus estimating the impact of malnutrition and the subsequent loss in muscle mass during treatment.

Based on the reported results, we believe that, in addition to investigating sarcopenia at baseline, monitoring patients at higher risk of developing sarcopenia during RT should be a component of future clinical practice for a tailored approach to patients. To the best of our knowledge, this is the first study to demonstrate the impact of sarcopenia during RT in the H&N setting [62,63,64].

Moreover, to facilitate the individuation of higher-risk patients, the validation of biomarkers associated with malnutrition or inflammatory status would be of fundamental help. In our study, a significant correlation was found between on-RT sarcopenia and reduced baseline hemoglobin values. This finding confirms literature data, considering the association between anemia and worse oncologic outcomes, often reported in previous published studies [65].

The Neutrophil-to-lymphocyte ratio (NLR), using a cut-off of three derived from a previously published study of our group in the OPC setting [21], was confirmed as significantly associated with worse oncological outcomes in our cohort as well, while association was not found between NLR and sarcopenia.

In this regard, one hypothesis is to consider NLR and sarcopenia as independently impacting the prognosis of the patients. Prospective studies with higher sample size are needed for the investigation of inflammatory status in relation to malnutrition and sarcopenia.

In our study, no association between sarcopenia and worse RT toxicities was reported, probably because of the limited sample size, or also because of the intensive clinical support (weekly physical examination) that is offered to patients at our institution to enable RT treatments to be better tolerated. However, considering that even severe CTCAE toxicities were reported in similar proportion between patients become sarcopenic during RT and never sarcopenic patients, probably the malnutrition responsible for the onset of sarcopenia during RT depends not only on the toxicities collected in our study, such as mucositis, dysphagia, and xerostomia, but also on other important symptoms, such as dysgeusia and nausea, often described during RT as impacting dramatically QoL [47]. In fact, despite the advantages of VMAT and Intensity Modulated RT (IMRT) IMRT techniques over conventional RT a series of severe acute and late toxicities are still experienced, prompting the future the implementation of additional innovative and sophisticated RT techniques, such as Proton therapy (PT) [66]. Indeed, some studies have shown that PT in H&N patients reduces not only late toxicities, but also acute toxicities during the treatment itself [66]. This hypothesis appears to be particularly promising, considering the increasing implementation of PT for several H&N tumors, especially for HPV-associated oropharyngeal carcinomas with long life expectancy in deintensification studies [67].

The current study is not exempt from limitations. Firstly, the retrospective nature of the study and the limited sample size. Secondly, the differences in treatment (RT schedules and systemic treatments) may have constituted a bias in the statistical analysis. Finally, for both models tested, thresholds for sarcopenia were considered as proposed by previous studies, which could not be optimally applicable to our subpopulation [68]. The homogeneous sample size representing a rare disease, the long follow-up, the application of different contouring methodologies and the assessment of sarcopenia during treatment represent the main strengths of the present analysis. Further studies are warranted in order to identify distinct cut-offs for sarcopenia in HPV-associated OPC setting, as well as consistent biomarkers correlating with inflammatory status and malnutrition, in order to encourage new personalized approaches within these patients [24,69,70].

## 5. Conclusions

Sarcopenia is often reported in several malignancies, especially in the case of H&N carcinomas, as associated with worse oncological outcomes. Besides the identification of baseline sarcopenia, the early detection of patients at higher risk of developing sarcopenia during RT would be essential as confirmed in the current study. In the specific setting of HPV-associated OPC, it is also mandatory to continue research aimed at identifying better tolerated therapeutic strategies with fewer late toxicities, to improve the QoL of these young, fit patients with long life expectancy. In this promising context, a tailored approach with earlier nutritional support to prevent sarcopenia during RT could improve the prognosis and the QoL of OPC patients.

## Figures and Tables

**Table 1:** Patients’ cohort characteristics.

Variables	Median (IQR)
Age at RT (years)	60 (53–67)
Hb at baseline (g/dL)	14.2 (13.2–15.3)
CCI	2 (2–3)
BMI (Kg/m^2^)	
baseline	26.01 (23.48–28.33)
End of RT	23.77 (21.70–25.55)
Lumbar_SMI from MM_CSA [cm^2^/m^2^]	
baseline	59.51 (39.57–51.60)
50 Gy	51.95 (46.38–57.18)
Lumbar_SMI from C3_CSA [cm^2^/m^2^]	
baseline	46.59 (66.16–89.92)
50 Gy	44.86 (36.42–49.40)
	*n* (%) N = 116
Sex	
Female	35 (30.2)
Male	81 (69.8)
Stage	
I	1 (0.9)
II	4 (3.4)
III	23 (19.8)
IV A	75 (64.6)
IV B	13 (11.3)
Tobacco smoking history	
Yes	64 (55.2)
No	33 (28.4)
Missing	19 (16.4)
Alcohol history	
Yes	48 (41.4)
No	43 (37.1)
Missing	25 (21.5)
HPV/p16 status	
Negative	7 (6.0)
Positive	86 (74.1)
Missing	23 (19.9)
KPS (%)	
<100	20 (17.2)
=100	96 (82.8)
Therapy	
RT	11 (9.5)
CRT	105 (90.5)
NLR at baseline	
<3	50 (43.1)
≥3	62 (56.9)
Nasogastric tube	
Yes	14 (12.1)
No	102 (87.9)
PEG	
Yes	12 (10.3)
No	104 (89.7)

List of abbreviations: BMI: Body mass index; CCI: Charlson Comorbidity Index; HB: Hemoglobin; HPV: Human Papilloma Virus; KPS: Karnofsky Performance Scale; NLR: Neutrophil-to-lymphocyte ratio; PEG: percutaneous endoscopic gastrostomy; RT: Radiotherapy.

**Table 2:** Results of multivariable Cox proportional hazard models related to PFS and OS (sarcopenia assessment based both on MM-CSA and C3-CSA).

Variable	MM-CSA		C3-CSA	
	PFS HR (CI 95%), *p* Value	OS HR (CI 95%), *p* Value	PFS HR (CI 95%), *p* Value	OS HR (CI 95%), *p* Value
Sarcopenia at 50 Gy Yes vs. No	2.52 (1.09–5.83), 0.03	2.69 (0.99–7.28), 0.05	1.99 (0.88–4.50), 0.097	1.03 (0.35–3.09), 0.95
Sex M vs. F	6.92 (2.13–22.5), 0.001	8.57 (2.01–36.5), 0.004	4.12 (1.65–10.3), 0.002	8.54 (2.00–36.4), 0.004
KPS <100 vs. = 100	3.63 (1.23–10.7), 0.02	-	-	3.91 (1.23–12.4), 0.02
Nasogastric tube Yes vs. No	2.71 (1.21–6.10), 0.01	7.69 (2.90–20.3), <0.001	3.02 (1.45–6.27), 0.003	3.55 (1.45–8.67), 0.005
Hb at baseline Continuous variable	0.72 (0.56–0.92), 0.008	0.63 (0.47–0.84), 0.001	0.72 (0.58–0.90), 0.004	0.66 (0.50–0.88), 0.004
NLR at baseline ≥3 vs. <3	2.67 (1.19–5.97), 0.02	-	-	3.92 (1.48–10.4), 0.006
CCI Continuous variable	-	2.10 (1.28–3.46), 0.003	-	-
Alcohol Yes vs. no	-	-	-	6.25 (1.24–29.2), 0.02

List of abbreviations: CCI: Charlson Comorbidity Index; HB: Hemoglobin; KPS: Karnofsky Performance Scale; NLR: Neutrophil-to-lymphocyte ratio; OS: Overall survival; PFS: Progression-free survival.

**Table 3:** Published retrospective studies on the association of sarcopenia with oncological and toxicity outcomes in patients with H&N carcinoma.

Author	Treatment Period	Total Patients	Follow-Up (Months)	Stage	RT Schedule	Sarcopenia Assessment	Proportion of Sarcopenic Patients	Sarcopenia Associations with Outcome	Sarcopenia Association with Toxicity
Ganju et al. [25], 2019	2012–2016	246 H&N (all sites)	35.1	Advanced 100%	60–70 Gy (IMRT) + ChT (also after primary surgery)	C3-CSA	34%	worse OS and DFS	/
van Rijn-Dekker et al. [29], 2020	2007–2016	750 H&N (all sites)	24	Early 24% Advanced 76%	70 Gy (3D, IMRT, VMAT) +/− ChT	C3-CSA	/	worse OS and DFS	late xerostomia and dysphagia
Cho et al. [49], 2018	2006–2015	221 H&N (all sites)	30	Advanced 100%	70 or less Gy (3D, IMRT) +/Cht	L3-CSA	48%	worse OS and DFS	/
Shodo et al. [47], 2021	/	41 H&N (all sites)	30	Early 12% Advanced 88%	70 Gy (3D, IMRT) + ChT	L3-CSA	27%	worse 2 years disease specific survival	/
Karavolia et al. [35], 2022	2007–2018	977 H&N (all sites)	/	Early 47% Advanced 53%	70 Gy (3D, IMRT, IMPT) +/− ChT	C3-CSA	25%	/	acute ≥ 3 dysphagia
Nagpal et al. [46], 2021	2016–2019	300 H&N (all sites)	24	Advanced 100%	70 Gy (IMRT, VMAT) + ChT	C3-CSA	/	worse DFS	more toxicities
Jin et al. [45], 2022	2017–2019	52 H&N (all sites)	/	Early 29% Advanced 71%	70 Gy (IMRT) +/− ChT	C3-CSA	/	worse local PFS	acute ≥ 3 toxicities
Thureau et al. [48], 2022	2014–2018	243 H&N (all sites)	36	Early 28% Advanced 72%	66–70 Gy (IMRT, VMAT) +/− ChT	L3-CSA	36.70%	worse OS and DFS	/
Bergamaschi et al., Current Study	2012–2019	118 OPC	61.2	Early 5% Advanced 95%	70 Gy (VMAT) +/− ChT	MM-CSA and C3-CSA	21% and 0% at baseline—36% and 13% at 50 Gy	worse OS and PFS with sarcopenia onset during RT	/

List of abbreviations: ChT: chemotherapy; DFS: Disease-free survival; H&N: Head & Neck; IMRT: Intensity-modulated radiation therapy; OPC: oropharynx cancer; OS: Overall survival; PFS: Progression-free survival; RT: Radiotherapy; VMAT: Volumetric modulated arc therapy; 3D: Three Dimensional Conformal Radiation Therapy.

## Data Availability

Data supporting the findings of this study are available upon reasonable request from the corresponding authors.

## Supplementary Materials

The following supporting information can be downloaded at: https://www.mdpi.com/article/10.3390/cancers15030723/s1, Figure S1: KM curves for PFS (top) and OS (bottom) of sarcopenic patients and not at baseline, based on MM-CSA and the formula validated by Prado et al.; Table S1: Sarcopenic condition at baseline and associations with patient characteristics; Table S2: Results of multivariable analysis for PFS and OS; Table S3: Sarcopenic onset during RT and associations with patients characteristics; Table S4: Sarcopenic onset during RT and associations with blood parameters, both at baseline and at the end of RT; Table S5: Sarcopenic onset during RT and associations activation of nutritional counseling during RT; Table S6: Sarcopenic onset during RT and associations with blood parameters, both at baseline and at the end of RT; Table S7: Associations between becoming sarcopenic at 50 Gy and blood parameters collected at baseline; Table S8: Sarcopenic onset during RT and associations activation of nutritional counseling during RT; Table S9: Results of multivariable Cox proportional hazard models related to PFS and OS (muscle loss considered as continuous variable).

## Abbreviations

AJCC: American Joint Committee on Cancer; C3: third cervical vertebra; CCI: Charlson Comorbidity Index; ChT: chemotherapy; CRT: combined treatment of radiotherapy and chemotherapy; CSA: cross-sectional area (CSA); CT: computed tomography; CTCAE: Common Terminology Criteria for Adverse Events; ECE: extracapsular extension; Gy: Gray; H&N: head and neck; HPV: human papilloma virus; IMRT: intensity modulated radiation therapy; IQR: interquartile range; IUCC: Union for International Cancer Control; KM: Kaplan–Meier; KPS: Karnofsky Performance Status; L3: third lumbar vertebra; MM: masticatory muscles; NLR: neutrophil-to-lymphocyte ratio; NTCP: normal tissue complication probability; OARs: organs at risk; OS: overall survival; PEG: percutaneous endoscopic gastrostomy; PFS: progression-free survival; PLR: platelet-to-lymphocyte ratio; PT: Proton therapy; QoL: quality of life; Rev-CT: revaluation CT; RT: radiotherapy; SIB: simultaneous integrated boost; Sim-CT: simulation CT; SMI: Skeletal Muscle Index; PF: docetaxel, cisplatin, and 5-fluorouracil; VMAT: volumetric modulated arc therapy.
