# Supplementary material for: Prognostic Impact of Sarcopenia’s Occurrence during Radiotherapy in Oropharyngeal Cancer Patients
Source: Cancers (Basel). 2023 Jan 24;15(3):723. doi: 10.3390/cancers15030723 (PMC9913355; doi:10.3390/cancers15030723)
Supplement: Supplementary file 1 [file cancers-15-00723-s001.zip › cancers-2130245-supplementary.pdf]

cancers

MDPI

Supplementary Material: Prognostic Impact of Sarcopenia's Occurrence During Radiotherapy in Oropharyngeal Cancer Patients

Luca Bergamaschi, Giulia Marvaso, Mattia Zaffaroni, Maria Giulia Vincini, Oriana D'Ecclesiis, Stefania Volpe, Annamaria Ferrari, Stefano Filippo Zorzi, Maria Cossu Rocca, Annarita Sabbatini, Giulia Cannillo, Emanuela Zagallo, Anna Starzyńska, Mohssen Ansarin, Federica Cattani, Sara Gandini, Roberto Orecchia, Daniela Alterio and Barbara A. Jereczek-Fossa

1. Sarcopenic condition at baseline based on CSA-MM.

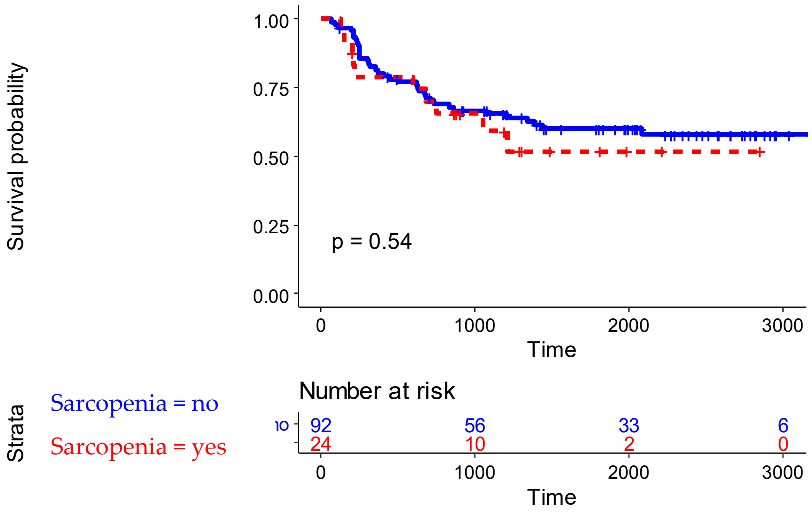

(a)

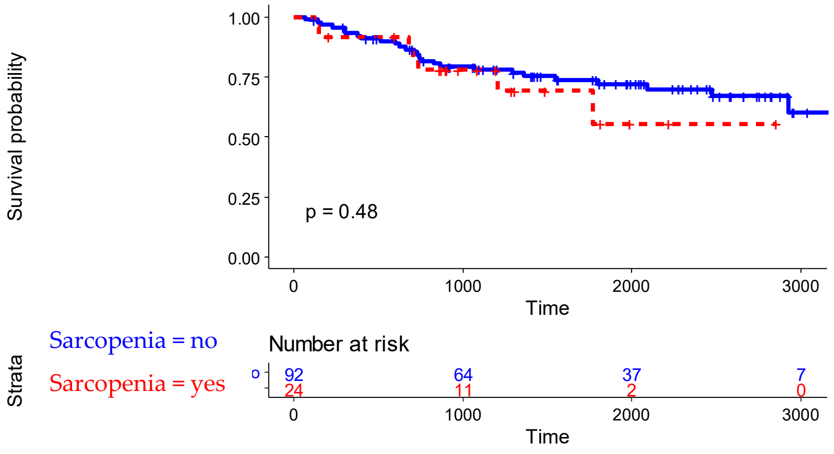

(b)

Figure S1. KM curves for PFS (a) and OS (b) of sarcopenic patients and not at baseline, based on MM-CSA and the formula validated by Prado et al.[47].

Table S1. Sarcopenic condition at baseline and associations with patient characteristics.

Variable	Overall (n = 116)	Sarcopenic Patients at Baseline (n = 24, 20.7%)	Non Sarcopenic Patients at Baseline (n = 92, 79.3%)	p-value
Age at RT, median (IQR)	60 (53–67)	60.5 (54.2–67.5)	60 (53–66.2)	0.83
Sex				
Female, *n* (%)	35 (30.2)	0 (0)	35 (38)	0.0008
Male, *n* (%)	81 (69.8)	24 (100)	57 (62)	
Stage				
I, *n* (%)	1 (0.9)	0 (0)	1 (1.1)	0.70
II, *n* (%)	4 (3.4)	0 (0)	4 (4.3)	
III, *n* (%)	23 (19.8)	4 (16.7)	19 (20.6)	
IV A, *n* (%)	75 (64.6)	18 (75)	57 (61.9)	
IV B, *n* (%)	13 (11.3)	2 (8.3)	11 (12.1)	
Smoking history				
Yes, *n* (%)	64 (55.2)	11 (45.8)	53 (57.6)	0.06
No, *n* (%)	33 (28.4)	12 (50.0)	21 (22.8)	
Missing, *n* (%)	19 (16.4)	1 (4.2)	18 (19.6)	
Alcohol history				
Yes, *n* (%)	48 (41.4)	14 (58.3)	34 (36.9)	0.35
No, *n* (%)	43 (37.1)	8 (33.3)	35 (38.0)	
Missing, *n* (%)	25 (21.5)	2 (8.4)	23 (25.1)	
HPV/p16 status				
Negative, *n* (%)	7 (6.0)	4 (16.7)	3 (3.3)	0.04
Positive, *n* (%)	86 (74.1)	17 (70.8)	69 (75)	
Missing, *n* (%)	23 (19.9)	3 (12.5)	20 (21.7)	
KPS				
<100, *n* (%)	20 (17.2)	6 (25)	14 (15.2)	0.37
=100, *n* (%)	96 (82.8)	18 (75)	78 (84.8)	
Therapy				
RT, *n* (%)	11 (9.5)	3 (12.5)	8 (8.7)	0.69
CRT, *n* (%)	105 (90.5)	21 (87.5)	84 (91.3)	
NLR				
<3	50 (43.1)	6 (25)	44 (47.8)	0.05
⩾3	62 (56.9)	18 (75)	44 (52.2)	
Nasogastric tube				
Yes	14 (12.1)	24 (100)	78 (84.8)	0.06
No	102 (87.9)	0 (0)	14 (15.2)	
PEG				
Yes	12 (10.3)	19 (79.2)	85 (92.4)	0.07
No	104 (89.7)	5 (20.8)	7 (7.6)	
Hb at baseline, median (IQR)	14.2 (13.2–15.3)	14.1 (13.5–15.0)	14.2 (13.0–15.4)	0.98

Table S2. Results of multivariable analysis for PFS and OS.

Variable	PFS		OS	
	**HR (CI 95%)**	***p*-value**	**HR (CI 95%)**	***p*-value**
Sarcopenia at baseline<br>Yes vs. No	0.86 (0.38–1.94)	0.72	0.62 (0.20–1.98)	0.42
Sex<br>M vs. F	5.25 (1.97–14.0)	0.0009	11.5 (2.64–49.9),	0.001
KPS<br><100 vs 100	2.79 (1.28–6.07)	0.01	6.20 (2.14–18.0)	0.0008
Nasogastric tube<br>Yes vs. No	2.20 (0.97–4.97)	0.057	2.69 (1.06–6.86)	0.04
Hb at baseline<br>Continuous variable	0.80 (0.64–0.99)	0.045	0.71 (0.54–0.91)	0.008
PEG<br>Yes vs. No	2.90 (1.19–6.89)	0.02	-	
NLR at baseline<br>⩾3 vs. <3	-		4.60 (1.72–12.3)	0.002
Alcohol<br>Yes vs. No	-		5.66 (1.52–21.1)	0.01

2. Sarcopenia onset at 50 Gy based on CSA-MM

Table S3. Sarcopenic onset during RT and associations with patients characteristics.

Variable	Overall (*n* = 92)	Sarcopenic Patients at 50 Gy (Not at Baseline) (*n* = 16, 17.4%)	Never Sarcopenic Patients (*n* = 69, 82.6%)	*p*-value
Age at RT, median (IQR)	60 (53–66.2)	61.5 (51.5–65.2)	60 (53–66)	0.90
Sex				
Female, *n* (%)	35 (38)	1 (6.2)	32 (46.4)	0.007
Male, *n* (%)	57 (62.0)	15 (93.8)	37 (53.6)	
Stage				
I, *n* (%)	1 (1.1)	0 (0)	1 (1.4)	0.54
II, *n* (%)	4 (4.3)	1 (6.2)	3 (4.3)	
III, *n* (%)	19 (20.6)	5 (31.2)	14 (20.3)	
IV A, *n* (%)	57 (61.9)	8 (50)	44 (63.8)	
IV B, *n* (%)	11 (12.1)	2 (12.6)	7 (10.2)	
Smoking status				
Yes, *n* (%)	53 (57.6)	7 (43.7)	44 (63.8)	0.22
No, *n* (%)	21 (22.8)	4 (25)	15 (21.7)	
Missing, *n* (%)	18 (19.6)	5 (31.3)	10 (14.5)	
Alcohol status				
Yes, *n* (%)	34 (36.9)	9 (56.2)	24 (34.8)	0.06
No, *n* (%)	35 (38.0)	2 (12.5)	31 (44.9)	
Missing, *n* (%)	23 (74.9)	5 (68.7)	14 (20.3)	
HPV/p16 status 16				
Negative, *n* (%)	3 (3.3)	3 (18.7)	0 (0)	0.007
Positive, *n* (%)	69 (75)	10 (62.5)	54 (78.3)	
Missing, *n* (%)	20 (21.7)	3 (18.8)	15 (21.7)	
KPS				
<100, *n* (%)	14 (15.2)	1 (6.2)	12 (17.4)	0.46
=100, *n* (%)	78 (84.8)	15 (93.8)	57 (82.6)	
Therapy				
RT, *n* (%)	8 (8.7)	0 (0)	8 (11.6)	0.21
CRT, *n* (%)	84 (91.3)	16 (100)	61 (88.4)	
NLR				
<3	44 (47.8)	12 (75)	29 (42.0)	0.05
⩾3	44 (47.8)	4 (25)	36 (52.2)	
Missing	4 (4.3)	0 (0)	4 (5.8)	
Nasogastric tube				
Yes	14 (15.2)	2 (12.5)	12 (17.4)	0.74
No	78 (84.8)	14 (87.5)	57 (82.6)	
PEG				
Yes	7 (7.6)	0 (0)	6 (8.7)	0.35
No	85 (92.4)	16 (100)	63 (91.3)	
Hb at baseline, median (IQR)	14.2 (13.05–15.4)	15.1 (12.1–15.6)	14.1 (13.1–15.4)	0.41
CCI, median (IQR)	2 (2–3)	2(2–2)	2 (2–3)	0.42

Table S4. Sarcopenic onset during RT and associations with blood parameters, both at baseline and at the end of RT.

	Variable	Sarcopenic Patients at 50 Gy (*n* = 16, 17.4%) Median (IQR)	Never Sarcopenic Patients (*n* = 69, 82.6%) Median (IQR)	*p*-value
Baseline	NLR	2.61 (2.44–3.07)	3.21 (2.14–3.91)	0.22
	Hb	14.1 (13.5–15.0)	14.2 (13.0–15.4)	0.98
	PLR	132.5 (120.2–137.9)	163.9 (120.3–194.6)	0.11
	Platelets	212.0 (169.7–240.5)	234.0 (199.0–275.0)	0.12
	Monocytes	0.61 (0.53–0.74)	0.59 (0.43–0.78)	0.41
End of RT	NLR	9.51 (7.75–14.7)	12.6 (7.72–17.0)	0.56
	Hb	11.9 (10.8–12.4)	11.9 (11.3–12.9)	0.41
	PLR	485.9 (342.7–613.1)	582.0 (441.2–895.1)	0.08
	Platelets	164.5 (156.7–200.5)	214.0 (153.5–262.0)	0.05
	Monocytes	0.62 (0.47–0.68)	0.52 (0.40–0.73)	0.58

Table S5. Sarcopenic onset during RT and associations activation of nutritional counseling during RT.

Variable	Sarcopenia (yes) (*n* = 16)	Sarcopenia (no) (*n* = 69)	*p*-value
			0.75
Counseling (yes) (*n* = 21)	3 (14.3)	18 (85.7)	
Counseling (no) (*n* = 64)	13 (20.3)	51 (79.7)	

3. Sarcopenia onset at 50 Gy based on CSA-C3

Table S6. Sarcopenic onset during RT and associations with blood parameters, both at baseline and at the end of RT.

Variable	Overall (*n* = 108)	Sarcopenic Patients at 50 Gy (Not at Baseline) (*n* = 14, 13.0%)	Never Sarcopenic Patients (*n* = 94, 87.0%)	*p*-value
Age at RT, median (IQR)	60 (53–67)	70.5 (64.5–76.7)	59 (52–64)	<0.001
Sex				
Female, *n* (%)	33 (30.5)	2 (14.3)	31 (33.0)	0.20
Male, *n* (%)	75 (69.5)	12 (85.7)	63 (67.0)	
Stage				
I, *n* (%)	1 (0.9)	0 (0)	1 (1.1)	
II, *n* (%)	4 (3.7)	1 (7.1)	3 (3.2)	
III, *n* (%)	23 (21.3)	2 (14.3)	21 (22.3)	0.74
IV A, *n* (%)	69 (63.9)	10 (71.5)	59 (62.8)	
IV B, *n* (%)	11 (10.2)	1 (7.1)	10 (89.4)	
Smoking status				
Yes, *n* (%)	62 (57.4)	8 (57.1)	53 (56.4)	
No, *n* (%)	31 (28.7)	4 (28.6)	27 (28.7)	0.99
Missing, *n* (%)	16 (13.9)	2 (14.3)	14 (14.9)	
Alchol status				
Yes, *n* (%)	47 (43.5)	6 (42.8)	41 (43.6)	
No, *n* (%)	40 (37.0)	4 (28.6)	36 (38.3)	0.64
Missing, *n* (%)	21 (19.5)	4 (28.6)	17 (18.1)	
HPV status 16				
Negative, *n* (%)	6 (5.5)	1 (7.1)	5 (5.3)	
Positive, *n* (%)	81 (75.0)	8 (57.1)	73 (77.6)	0.22
Missing, *n* (%)	21 (19.5)	5 (64.2)	16 (17.1)	
KPS				0.23

<100, *n* (%)	18 (16.7)	4 (28.6)	14 (14.9)	
=100, *n* (%)	90 (83.3)	10 (71.4)	80 (85.1)	
Therapy<br>RT, *n* (%)<br>CRT, *n* (%)	10 (9.3)<br>98 (90.7)	3 (21.4)<br>11 (78.6)	7 (7.5)<br>87 (92.5)	0.12
NLR				
<3	47 (43.5)	4 (28.6)	43 (45.7)	0.41
⩾3	57 (52.8)	9 (64.3)	48 (51.1)	
Missing	4 (3.7)	1 (7.1)	3 (3.2)	
Nasogastric tube				
Yes	14 (13.0)	4 (28.6)	10 (10.6)	0.06
No	94 (87.0)	10 (71.4)	84 (89.4)	
PEG				
Yes	11 (10.2)	3 (21.4)	8 (8.5)	0.15
No	97 (89.8)	11 (78.6)	86 (91.5)	
HB at baseline, median (IQR)	14.2 (13.2–15.3)	13.4 (12.6–14.1)	14.5 (13.3–15.5)	0.03
CCI median (IQR)	2 (2–3)	2(2–2)	2 (2–3)	0.76

Table S7. Associations between becoming sarcopenic at 50 Gy and blood parameters collected at baseline.

	Variable	Sarcopenic Patients at 50 Gy (*n* = 14, 13%) Median (IQR)	Never Sarcopenic Patients (*n* = 94, 87%) Median (IQR)	*p*-value
Baseline	NLR	3.36 (2.97–4.32)	3.04 (2.16–3.87)	0.08
	Hb	13.4 (12.6–14.1)	14.5 (13.3–15.5)	0.03
	PLR	174.3 (127.5–247.6)	147 (121.7–192.7)	0.28
	Platelets	218 (169–265)	239 (202–286)	0.33
	Monocytes	0.67 (0.55–0.73)	0.61 (0.47–0.74)	0.61
End of RT	NLR	13.2 (10.4–16.1)	11.6 (7.55–19.5)	0.57
	Hb	12 (11.07–12.5)	11.9 (11.1–12.8)	0.80
	PLR	496.5 (424.9–1005.0)	568.3 (427.5–938.4)	0.83
	Platelets	222.5 (178.2–256.2)	205.5 (157.0–258.5)	0.43
	Monocytes	0.60 (0.51–0.75)	0.54 (0.39–0.74)	0.40

Table S8. Sarcopenic onset during RT and associations activation of nutritional counseling during RT.

Variable	Sarcopenia (yes) (*n* = 14)	Sarcopenia (no) (*n* = 94)	*p*-value
			0.02
Counseling (yes) (*n* = 31)	8 (25.8)	23 (79.2)	
Counseling (no) (*n* = 77)	6 (7.8)	71 (92.2)	

Table S9. Results of multivariable Cox proportional hazard models related to PFS and OS (muscle loss considered as continuous variable).

Variable	MM-CSA		C3-CSA	
	PFS HR (CI 95%), *p*-value	OS HR (CI 95%), *p*-value	PFS HR (CI 95%), *p*-value	OS HR (CI 95%), *p*-value
Sarcopenia at 50 Gy continuous variable	0.93 (0.87–0.99), 0.02	0.89 (0.82–0.96), 0.005	1.00 (0.94–1.06), 0.99	1.03 (0.95–1.11), 0.51
Sex M vs. F	13.3 (3.43–51.3), 0.0002	16.9 (3.87–73.8), 0.0002	4.40 (1.34–14.4), 0.01	6.94 (1.45–33.09), 0.01
KPS	3.82 (1.28–11.4), 0.02	-	-	4.34 (1.43–13.1), 0.009

<100 vs. = 100				
Nasogastric tube<br>Yes vs. No	2.69 (1.19–6.08), 0.02	9.69 (3.46–27.1), <0.001	3.18 (1.51–6.70), 0.002	3.77 (1.55–9.14), 0.003
Hb at baseline<br>Continuous variable	0.70 (0.55–0.91), 0.008	0.57 (0.41–0.77), 0.0004	0.70 (0.57–0.86), 0.0008	0.66 (0.50–0.88), 0.004
NLR at baseline<br>⩾3 vs. <3	2.26 (1.04–4.90), 0.04	-	-	4.05 (1.52–10.8), 0.005
CCI<br>Continuous variable	-	2.57 (1.54–4.30), 0.0003	-	-
Alcohol<br>Yes vs. no	-	-	-	6.02 (1.30–27.9), 0.02
